# Pathogenesis and Host Response to A3B4 and A3B2 Infectious Bursal Disease Virus Strains in Chickens

**DOI:** 10.1155/tbed/6522916

**Published:** 2026-09-28

**Authors:** Anna Pikuła, Anna Lisowska, Justyna Opolska, Piotr Kubiś

**Affiliations:** ^1^ Department of Virology and Animal Viral Diseases, National Veterinary Research Institute, Al. Partyzantow 57, 24-100, Pulawy, Poland, nvri.gov.ng

**Keywords:** A3B4 genotype, host response, immunosuppression, infectious bursal disease virus (IBDV), pathogenesis, poultry health, transcriptomics, viral persistence

## Abstract

Recently, the identification of novel reassortants of infectious bursal disease (IBD) virus (IBDV) was reported. Interestingly, these strains competed with the previously dominant A3B2 lineage, becoming the prevalent lineage in Poland and disseminating to other countries in Europe. However, the specific virulence properties that facilitate their spreading were not elucidated. In this study, we compared the pathogenesis of the A3B4 Bug/03 and A3B2 75/11 IBDV strains in specific‐pathogen‐free (SPF) chickens, integrating viral replication dynamics, tissue tropism, immunosuppressive effects and host transcriptional responses. Both strains replicated efficiently in lymphoid and non‐lymphoid tissues, with peak viral loads in the bursa of Fabricius. While the A3B2 75/11 strain exhibited higher viral RNA levels at early time points, the A3B4 Bug/03 strain persisted longer in multiple tissues. Notably, the A3B4 Bug/03 strain induced earlier bursal atrophy despite comparable viral loads at 4 dpi, and was associated with heterogeneous growth performance, reflecting uneven flock development. Transcriptomic analysis revealed distinct host response profiles between strains. Infection with the A3B4 virus was characterised by the enrichment of metabolic and translational pathways, indicating cellular adaptation and metabolic reprogramming. In contrast, the A3B2 75/11 strain triggered a strong activation of immune and inflammatory pathways, including cytokine and chemokine signalling. Collectively, these findings demonstrate that IBDV pathogenicity is not solely determined by viral replication, but is critically shaped by host response mechanisms. The ability of A3B4 strains to modulate host pathways and persist in infected birds likely contributes to their epidemiological success and facilitates their continued spread in poultry populations.

## 1. Introduction

Infectious bursal disease (IBD), also known as Gumboro disease, is an immunosuppressive disorder that primarily affects young chickens [1]. The aetiological agent of the disease is the IBD virus (IBDV), a member of the family Birnaviridae and the genus *Avibirnavirus*. The viral genome consists of two segments of double‐stranded RNA. Segment A encodes four viral proteins, including the non‐structural protein VP5 and a precursor polyprotein that is further processed into the mature proteins VP2, VP3, and VP4 [2]. The capsid protein VP2 is responsible for virus–host interaction [3] and induction of neutralising antibodies [4]. Segment B encodes the viral RNA‐dependent RNA polymerase (VP1), which catalyses replication and transcription [5] and, together with VP3, contributes to initiation of translation [6].

IBDV has a significant impact on the poultry industry, causing increased mortality, enhanced susceptibility to secondary infection, poor flock uniformity, reduced feed efficiency, and vaccination failures [7]. However, the effectiveness of vaccination is influenced by several factors, including the level of maternally derived antibodies, improper timing of vaccination leading to an “immunity gap,” and the type of IBDV strains circulating in the field [8]. As a result, IBDV infections are widespread globally and continue to cause substantial economic losses.

Currently, the global epidemiological situation of IBDV is largely driven by the emergence of novel variants and very virulent and reassortant strains [9–11]. These viruses differ in virulence or antigenic properties, enabling them to evade the host immune response and replicate efficiently, thereby influencing the disease outcome.

Recently, the co‐existence of two divergent IBDV lineages was identified in the field in Poland: A3B4 strains, characterised by a vvIBDV‐derived segment A and a distinct B4 segment, and the A3B2 lineage [12]. Notably, A3B4 strains successfully competed with the previously dominant A3B2 lineage, becoming prevalent in Poland and spreading to other countries in Europe [13]. However, the mechanisms underlying the epidemiological fitness of the A3B4 lineage remain poorly understood. We hypothesised that A3B4 strains differ from A3B2 strains not only in replication dynamics but also in the nature of host responses, which may contribute to their epidemiological fitness.

Therefore, the present study aimed to compare the pathogenesis of A3B2 75/11 and A3B4 Bug/03 strains in specific‐pathogen‐free (SPF) chickens. The analysis encompassed multiple aspects of in vivo infection, including viral replication in various tissues, cloacal shedding, body weight gain, immunosuppressive effects, and host gene expression profiling using high‐throughput RNA sequencing (RNA‐seq).

## 2. Materials and Methods

### 2.1. Ethical Statement

All experimental procedures were reviewed and approved by the Local Ethical Commission in Lublin (Permit Number 37/2020 and 119/2022) and were conducted in accordance with the European Union Directive 2010/63/EU on the protection of animals used for scientific purposes.

### 2.2. Viruses

Two IBDV strains were used in this study: Bug/03/Poland/2003 (GenBank accession numbers: segment A: MT629830, segment B: MT629833) and 75/11/Poland/2011 (segment A: MT629832, segment B: MT629835), representing genotype A3B4 and A3B2, respectively, as previously characterised by Pikuła et al. [13]. Prior to infection, virus stocks were treated with chloroform as previously described [14] to inactivate potential enveloped viral concomitants. Viral titres were expressed as median embryo infective dose (EID_50_) and calculated using the Reed–Muench method.

### 2.3. Experimental Design

A total of 80 5‐week‐old SPF White Leghorn chickens (Valo BioMedia, Germany) were randomly allocated into three groups: groups 1 and 2 (*n* = 30 each) and group 3 (*n* = 20). Birds in groups 1 and 2 were inoculated intraocularly with 10^5^ EID_50_/bird of Bug/03 (A3B4) and 75/11 (A3B2), respectively. Chickens in group 3 received sterile saline and served as the negative control. Birds were housed in HEPA‐filtered isolators (Montair Andersen B.V., Holland) with ad libitum access to feed and water and monitored daily for 28 days. At 2, 4, 7, 14, 21 and 28 days post‐infection (dpi), five randomly selected birds from groups 1 and 2 and 3 from the control group were euthanized. Additionally, at 4 dpi, an extra two control birds were euthanised (*n* = 5 total) for transcriptomic (RNA‐seq) analysis. Cloacal swabs were collected prior to necropsy.

All birds were subjected to post‐mortem examination, and gross pathological lesions were recorded. Tissue samples (bursa of Fabricius, spleen, thymus, caecal tonsils, kidney, liver, duodenum and proventriculus) were collected and stored at −70°C for further analysis. At each time point, the body weight, as well as bursa and spleen weights, were recorded. The bursa‐to‐body weight ratio (B:BW) was calculated for each bird, and the bursa‐to‐body weight index (BBIX) was determined by dividing the individual B:BW ratio of each infected bird by the mean B:BW ratio of the corresponding control group at the same sampling point. BBIX values below 0.7 were considered indicative of bursal atrophy [15].

### 2.4. Transcriptome Sequencing and Analysis

Bursa of Fabricius tissues (*n* = 5 per group) collected at 4 dpi were used for transcriptomic analysis. The 4 dpi time point was selected prospectively to capture strain‐dependent host responses during the acute phase of infection. Immediately after collection, bursal samples designated for RNA‐seq were placed on ice, subsequently processed and aliquoted, and stored at −70°C until RNA extraction. Total RNA was extracted using the RNeasy Mini Kit (Qiagen, Germany) according to the manufacturer’s protocol. Approximately 30 mg of tissue was homogenised in 600 μL of RLT buffer supplemented with β‐merkaptoethanol using a FastPrep‐24 homogeniser (MP Biomedical, USA).

RNA concentration and purity were assessed using a NanoDrop 2000 spectrophotometer (Thermo Fisher Scientific), and RNA integrity was evaluated using a 2100 Bioanalyzer (Agilent Technology, USA) and the RNA 6000 nano Kit. Samples with an RNA integrity number (RIN) ≥6.5 were considered acceptable for further analysis. Library preparation (TruSeq Stranded mRNA LT Sample Prep Kit, Illumina, USA) and sequencing (NovaSeq 6000, Illumina, USA) were performed by Macrogen Europe B.V. (the Netherlands).

Raw 150 bp paired‐end reads were analysed by Data2Biology (Poznan, Poland). Quality control was performed using FastQC. Adapter trimming and quality filtering were carried out using BBduk2 (BBTools suite, v37.02). Reads mapping to chicken ribosomal RNA sequences (ENSEMBL release 108) were removed using Bowtie2. Filtered reads were aligned to the *Gallus gallus* reference genome (bGalGal1.mat.broiler.GRCg7b) using the STAR (v2.5.3a).

Gene and transcript expression levels were quantified using RSEM (v1.2.28). Differential gene expression analysis was performed using DESeq2, and differentially expressed genes (DEGs) were defined using an adjusted *p*‐value <0.05 |log_2_ fold change| >2 [16]. Functional enrichment analysis was conducted using g:GOSt tool (g:Profiler) to identify over‐represented gene ontology terms and the KEGG pathways (threshold *p* < 0.05).

### 2.5. Validation of RNA‐Seq Results

To validate the RNA‐seq data, five DEGs were selected for RT‐qPCR analysis: IFIT5, CCLL4, CD72, TNFRSF13C and CCL4. Primers and probe sequences are provided in Table 1. Gene expression was quantified using the QuantiTect Probe RT‐PCR Kit (Qiagen, Germany) on a 7500 Real‐Time System (Applied Biosystems, USA). Each sample was analysed in duplicate. Relative gene expression levels were calculated using the 2^−ΔΔCt^ method, normalised to ACTB as the reference gene, and expressed as log_2_ fold change.

**Table 1:** Primers and probes used in the study for validation of RNA‐seq.

Target gene	Sequence (5′–3′)	References
ACTB	F: CATCCTCACCCTGAAGTACC R: GCTCATTGTAGAAGGTGTGG Probe: CACGGCATCGTCACCAACTG	Vora et al. [17]
IFIT5	F: AAAAGAAGGCAAATCATGAGTAC R: TGATCCTCTATTGATTCTTCCAGAC Probe: AATTCCTTGAAGAACTCCCTGCTGC	Cong et al. [18]
CCLL4	N/A	Thermo Fisher Gg03360167_m1
CD72	N/A	Thermo Fisher Gg03348391_g1
TNFRSF13C	N/A	Thermo Fisher Gg03357593_m1
CCL4	N/A	Thermo Fisher Gg03338617_m1

### 2.6. Sample Preparation

Cloacal swabs were suspended in 1 mL of 1x PBS, incubated for 1 h at room temperature, and centrifuged at 3500 *g* for 15 min at 4°C. A 10% (w/v) suspension of tissue samples was prepared in 1x PBS and processed under the same centrifugation conditions. Supernatants were collected and used for RNA extraction.

### 2.7. Viral Load Quantification by RT‐qPCR

Viral RNA was extracted using the IndiMag Pathogen Kit (Indical Bioscience, Germany) on an IndiMag 48 system (Indical Bioscience, South Korea), following manufacturer’s instructions. Quantification of IBDV RNA was performed using a previously described RT‐qPCR assay [19]. RNA transcripts corresponding to the VP2 gene of the 75/11 strain were generated and used as standards. A 1409 bp fragment was amplified using the SuperScript III One‐Step RT‐PCR System (Invitrogen, Thermo Fischer Scientific, Lithuania) and primers (VP2cds_F: 5′‐ATGGTTAGTAGAGATCAGACAAACG‐3′ and VP2cds_R: 5′‐ACCACCGGCACAGCTATCCT‐3′). RNA standards were prepared as described previously [20], treated with DNase I, and purified using the RNeasy Mini Kit (Qiagen, Germany). RNA concentration was measured using a NanoPhotometer Pearl (Implen, Germany) and converted to copy number using NEBioCalculator. Tenfold serial dilutions ranging from 10^8^ to 10^3^ copies/µL were used to generate standard curves. Viral loads in samples were calculated based on *C*_*t*_ values.

### 2.8. Immunosuppression Study

Forty one‐day‐old SPF chickens were randomly divided into four groups (G1–G4, *n* = 10 per group). Birds in G1 and G2 were inoculated intraocularly with 10^4^ EID_50_/bird of Bug/03 and 75/11 IBDV strains, respectively. Groups G3 and G4 served as vaccination and negative controls, respectively. At 14 days post‐infection, birds from G1–G3 were vaccinated with the Hitchner B1 strain vaccine against Newcastle disease. Two weeks later, blood samples were collected for the hemagglutination inhibition (HI) assay. Subsequently, all birds were challenged intramuscularly with a velogenic Newcastle disease virus strain chicken/Poland/Radom/70 (ICPI = 1.88) at a dose of 10^5^ EID_50_/bird. Birds were monitored daily for 14 days for clinical signs and mortality.

### 2.9. Statistical Analysis

Statistical analyses were performed using GraphPad Prism v.10.6.0 (GraphPad Software, USA). Viral RNA loads in the bursa of Fabricius were log_10_‐transformed prior to analysis and compared between virus strains at each time point using multiple unpaired *t*‐tests with Welch’s correction, followed by the Holm–Šídák correction for multiple comparisons. For tissues containing non‐detectable samples, viral RNA loads were analysed using multiple Mann–Whitney tests with the Holm–Šídák correction. Gene expression data, expressed as log_2_ fold change, were analysed using Brown–Forsythe and Welch one‐way ANOVA, followed by Dunnett’s T3 multiple‐comparison test for pairwise comparisons between experimental groups. BBIX values, spleen‐to‐body weight ratios, and body weights were analysed using ordinary two‐way ANOVA with group and time post‐infection as factors, followed by Šídák’s multiple‐comparison test for pairwise comparisons between groups within each time point. Body weight data were log_10_‐transformed prior to analysis. Survival was visualised using Kaplan–Meier curves. Differences were considered statistically significant at *p* < 0.05.

## 3. Results

### 3.1. Clinical Findings

A summary of the parameters analysed in the pathogenicity study is shown in Figures 1 and 2. During the animal trial, no clinical signs or post‐mortem lesions were observed in the control group. In contrast, from 2 days post‐infection (dpi), birds in groups 1 and 2 developed clinical signs, including depression, ruffled feathers and diarrhoea. Mortality reached 30% (*n* = 10) in the Bug/03‐infected group and 36.7% (*n* = 11) in the 75/11‐infected group. Due to the observed mortality, the experiment was terminated at 14 dpi. Clinical signs gradually subsided from day 5 post‐infection and were no longer observed at 7 dpi. Kaplan–Meier survival curves for all experimental groups are shown in Figure 2D.

**Figure 1:**
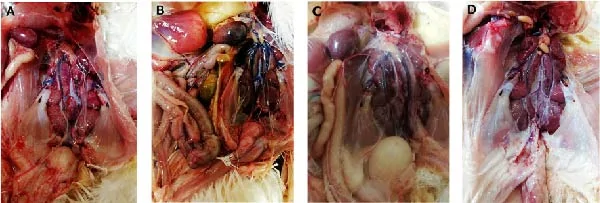
Gross lesions observed in chickens infected with the A3B4 Bug/03 strain (A, B) and the A3B2 75/11 strain (C, D). (A) Swollen kidneys, decreased size of the bursa of Fabricius and spleen with necrotic foci at 4 dpi. (B) Congested duodenum, bursal atrophy and necrotic spleen at 7 dpi. (C) Necrotic spleen and enlarged bursa at 2 dpi. (D) Swollen kidneys and bursal atrophy at 7 dpi.

**Figure 2:**
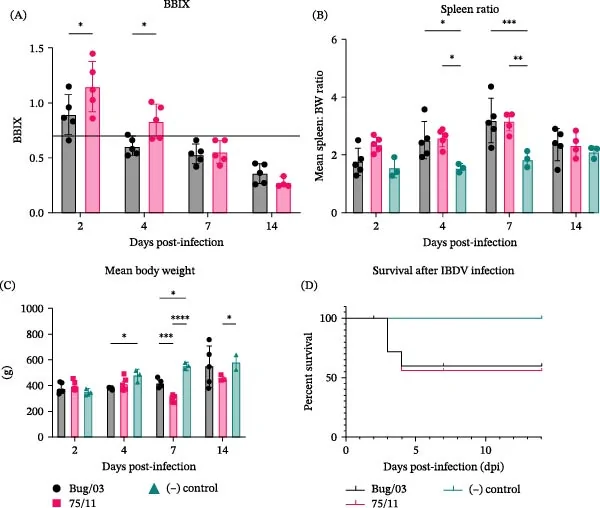
Assessment of the impact of IBDV infection on (A) bursa‐to‐body weight index (BBIX), (B) spleen‐to‐body weight ratio, (C) mean body weight and (D) survival following IBDV infection. Bars in (A–C) represent mean ± SD, (D) shows Kaplan–Meier survival curves. Statistical comparisons of body weight were performed using log_10_‐transformed values. Statistical significance is indicated as follows:  ^∗^ *p* < 0.05,  ^∗∗^ *p* < 0.01,  ^∗∗∗^ *p* < 0.001,  ^∗∗∗∗^ *p* < 0.0001.

At necropsy, several macroscopic lesions were observed in dead and euthanised birds, including enlarged, mottled spleens with necrotic foci, swollen kidneys and congested duodenum (Figure 1). Changes in bursal size differed between the infected groups. Although BBIX differed significantly between Bug/03‐ and 75/11‐infected chickens already at 2 dpi, the index remained above the threshold indicative of bursal atrophy (0.7) in both groups. At 4 dpi, BBIX decreased below 0.7 in the Bug/03 group, whereas it remained above this threshold in the 75/11 group. Bursal atrophy in the 75/11 group became evident at 7 dpi (Figure 2A).

A two‐way ANOVA revealed a significant interaction between virus strain and days post infection (*F*(3,31) = 3.480, *p* = 0.0275) and a significant effect of time (*F*(3,31) = 45.90, *p* < 0.0001). Šídák’s multiple‐comparisons test showed significantly higher BBIX values in the 75/11 group than in the Bug/03 group at 2 dpi (*p* = 0.0232) and 4 dpi (*p* = 0.0459), whereas no significant differences were observed at 7 or 14 dpi.

Spleen‐to‐body weight ratios were significantly higher than those of controls in both infected groups at 4 (Bug/03, *p* = 0.0207; 75/11, *p* = 0.0103) and 7 dpi (Bug/03, *p* = 0.00097; 75/11, *p* = 0.0012). No significant differences were detected at 2 or 14 dpi (Figure 2B).

To assess the impact of infection on growth performance, body weight was measured at each sampling point (Figure 2C). At 4 dpi, body weight was significantly lower in the Bug/03 group than in the control group (*p* = 0.0316). At 7 dpi, both infected groups had significantly lower body weights than controls (Bug/03, *p* = 0.0104; 75/11, *p* < 0.0001), and birds infected with 75/11 also had significantly lower body weights than Bug/03‐infected birds (*p* = 0.0007). At 14 dpi, body weight remained significantly lower in the 75/11 group than in controls (*p* = 0.0422). Mean body weight was lower than in controls by 104.1 and 134.4 g in the Bug/03 group at 4 and 7 dpi, respectively, and by 248.2 and 125.9 g in the 75/11 group at 7 and 14 dpi, respectively. Notably, a substantial variation in body weight was observed in the Bug/03 group at 14 dpi, ranging from 383 to 761 g.

### 3.2. Persistence of IBDV Strains in Tissues

Strain‐dependent differences in viral RNA loads were detected in the bursa of Fabricius, spleen, caecal tonsils and proventriculus. Viral RNA loads in the bursa of Fabricius were significantly higher in the 75/11 group at 2 and 7 dpi (*p* < 0.05 and *p* < 0.001, respectively). At 7 dpi, significantly higher viral RNA loads were also detected in the spleen and caecal tonsils of 75/11‐infected birds (*p* < 0.05). In contrast, at 14 dpi, viral RNA load in the spleen was significantly higher in the Bug/03 group (*p* < 0.05). No significant differences were observed in the thymus. Notably, Bug/03 remained detectable in all lymphoid tissues up to 14 dpi, whereas the 75/11 strain was detected at this time point only in the bursa of Fabricius (Figure 3). In non‐lymphoid tissues, a significantly higher viral RNA load was observed in the proventriculus of the 75/11 group at 7 dpi (*p* < 0.05); no significant differences were detected in the kidney, liver or duodenum (Figure 4).

**Figure 3:**
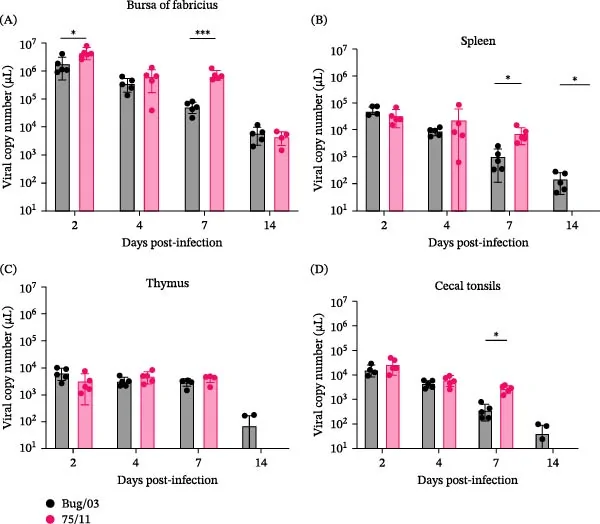
Mean viral load in lymphoid tissues of SPF chickens after infection with the A3B4 Bug/03 and A3B2 75/11 IBDV strains: (A) bursa of Fabricius, (B) spleen, (C) thymus, and (D) cecal tonsils. Bars represent mean ± SD, and individual points represent values obtained for individual birds. Statistical significance between the infected groups is indicated as follows:  ^∗^ *p* < 0.05,  ^∗∗∗^ *p* < 0.001.

**Figure 4:**
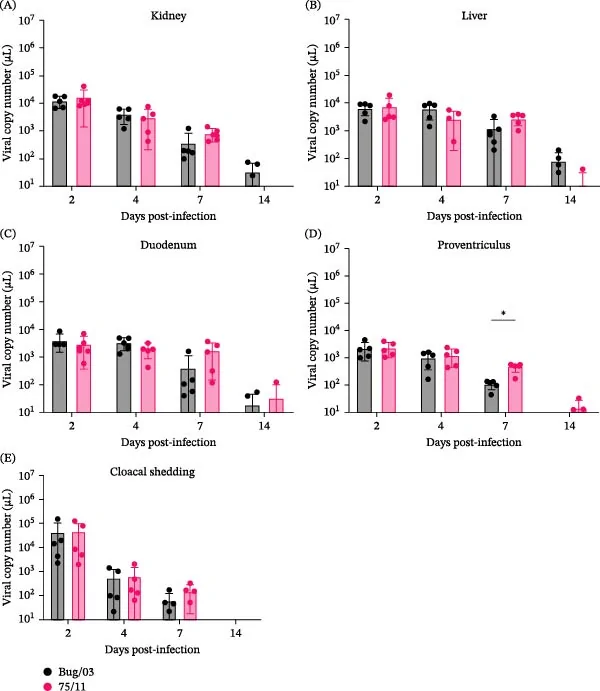
Mean viral load in non‐lymphoid tissues and cloacal shedding in SPF chickens after infection with the A3B4 Bug/03 and A3B2 75/11 IBDV strains: (A) kidney, (B) liver, (C) duodenum, (D) proventriculus, and (E) cloacal shedding. Bars represent mean ± SD, and individual points represent values obtained for individual birds. Statistical significance between the infected groups is indicated as follows: *p* < 0.05 and  ^∗^ *p* < 0.01.

#### 3.2.1. Viral Shedding

Viral shedding from the digestive tract is shown in Figure 4E. No significant differences were observed between the two infected groups.

#### 3.2.2. Immunosuppressive Effect

Detailed results are presented in Table 2. No clinical signs or mortality were observed in the vaccinated control group (G3) following the challenge with velogenic NDV. In contrast, birds in groups G1, G2 and G4 developed clinical signs including apathy, ruffled feathers, convulsions and paralysis between 2 and 8 days post‐challenge. A total of 18 birds died, including three that were euthanised according to humane endpoints. Serological analysis showed reduced seroconversion in IBDV‐infected groups (G1, G2), with only 30%–40% seropositive birds and low HI titres (16–32). In contrast, all birds in the G3 group seroconverted, with HI titres ranging from 16 to 128 (mean titre: 56).

**Table 2:** Summary of in vivo immunosuppression assessment results.

Experimental group	HI‐NDV results positive/total (titre range)	Symptoms	Mortality^a^	B‐BW ratio
G1 infected with Bug/03 and vaccinated ND	4/10 (16–32)^c^	7/10	5/10	0.93^b^
G2 infected with 75/11 and vaccinated ND	3/10 (16–32)^c^	10/10	3/10	0.80^b^
G3 vaccinated control	10/10 (16–128)^c^	0/10	0/10	5.87
G4 non‐vaccinated control	0/10 (<8)	10/10	10/10	N/A

^a^Including birds euthanised according to humane endpoints.

^b^Values significantly different from G3 (*p* < 0.01).

^c^HI titres are shown only for seropositive birds.

#### 3.2.3. Transcriptome Analysis

High‐throughput RNA‐seq generated a total of 454.8 million raw reads across all samples. Following quality trimming and adapter removal using BBDuk2, 406.7 million high‐quality reads were retained, representing 89.45% of the initial dataset, indicating high sequencing quality and efficient preprocessing.

Differential gene expression analysis identified 5479 and 5392 DEGs in chickens infected with the A3B4 Bug/03 and A3B2 75/11 strains, respectively, compared to the uninfected control (*p* < 0.05, |log_2_ fold change| >2). Among these, 3547 and 3462 genes were upregulated, while 1932 and 1930 genes were downregulated in the Bug/03 and 75/11 groups, respectively (Supporting Information 1: Table S1).

Functional enrichment analysis revealed extensive transcriptional reprogramming in both infected groups; however, distinct biological signatures were observed between the strains (Figure 5). GO analysis identified 413 significantly enriched terms in the Bug/03 group and 377 in the 75/11 group, spanning all three GO categories: biological processes (BPs), molecular function (MF) and cellular component (CC) (Supporting Information 2: Table S2).

**Figure 5:**
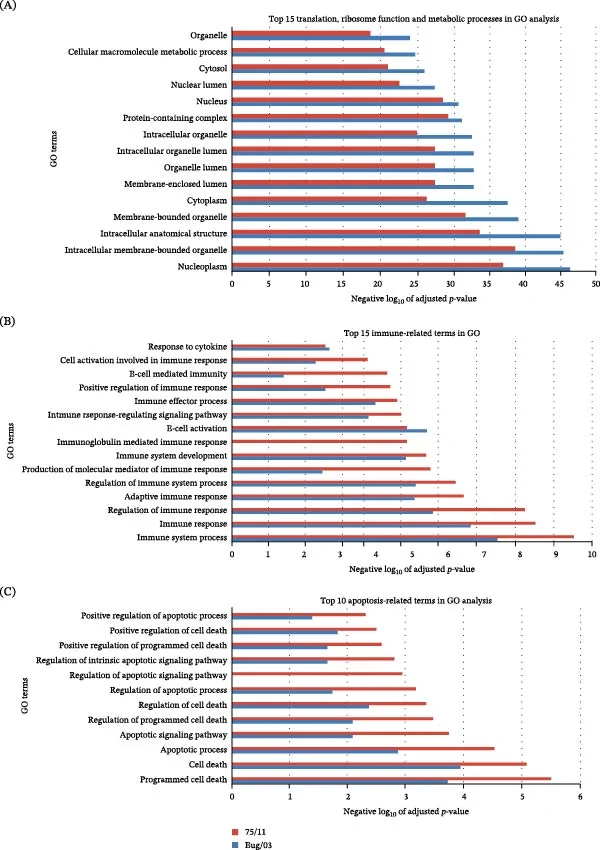
Results of gene ontology analysis for the most significant pathways in comparisons between the infected groups and the uninfected control group: (A) top 15 terms associated with translation, ribosome function, and metabolic processes; (B) top 15 immune‐related GO terms; and (C) top 10 apoptosis‐related GO terms. Bars represent the negative log_10_ of the adjusted *p*‐value for the A3B4 Bug/03 and A3B2 75/11 groups.

In the Bug/03‐infected group, enriched GO terms were predominantly associated with metabolic processes, RNA processing, translation and cell cycle regulation. MF categories were dominated by RNA binding and enzyme binding, while CC terms highlighted ribosome, mitochondrion and respiratory chain complexes. Consistently, KEGG pathway analysis indicated significant enrichment of ribosome, oxidative phosphorylation, RNA transport and spliceosome pathways.

In contrast, the transcriptomic profile of the 75/11‐infected group was characterised by enrichment of immune‐related processes, including regulation of immune response, adaptive immune response, B cell activation and apoptosis. MF categories included cytokine activity and receptor binding, while KEGG pathways such as cytokine–cytokine receptor interaction, chemokine signalling and apoptosis were significantly overrepresented.

Despite these differences, both groups shared a set of common BPs, including cellular stress response, DNA repair, apoptosis and translation, reflecting a general host response to IBDV infection.

A focused examination of apoptosis‐related genes revealed transcriptional changes in both infected groups. In Bug/03‐infected bursae, several genes associated with death‐receptor signalling, including FASLG, TNFRSF10B, CASP10 and CASP18, were upregulated. In the 75/11 group, CASP9, AIFM3, GADD45B and DAPK2 showed stronger induction than in the Bug/03 group. CASP3 was markedly downregulated in both infected groups, together with several genes with anti‐apoptotic functions, including BIRC5, BIRC7, API5 and AATF (Supporting Information 1: Table S1).

Direct comparison between the two infected groups identified a relatively small subset of 76 DEGs (40 upregulated and 36 downregulated; Supporting Information 1: Table S1, Bug03_vs_7511_DEG tab), suggesting that the majority of transcriptional responses were shared but differed in magnitude rather than direction. Notably, the 75/11 strain showed higher expression of genes associated with inflammatory and immune responses, including *CCL4*, *CSF2RA*, *CSF3R*, *S100A12*, and *MMP9*. In contrast, the Bug/03 strain exhibited higher expression of genes related to transcriptional regulation, RNA processing and cellular adaptation, such as *SOX5*, *ELAVL4*, *HSPA2* and *SFSWAP*.

### 3.3. Verification of DEGs Using RT‐qPCR

RNA‐seq results were validated using RT‐qPCR (Figure 6). CD72 and TNFRSF13C were significantly downregulated, whereas IFIT5 and CCLL4 were significantly upregulated in both infected groups compared with the control group. CCL4 expression was significantly increased in both infected groups and was significantly higher in the 75/11 group than in the Bug/03 group. Overall, RT‐qPCR results were consistent with the RNA‐seq data.

**Figure 6:**
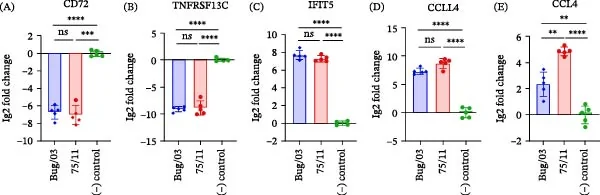
RT‐qPCR verification of selected differentially expressed genes (DEGs) in the bursa of Fabricius at 4 dpi: (A) CD72, (B) TNFRSF13C, (C) IFIT5, (D) CCLL4, and (E) CCL4. Bars represent mean ± SD, and individual points represent values obtained for individual birds. Gene expression is presented as log_2_ fold change relative to the uninfected control group. Statistical significance is indicated as follows:  ^∗∗^ *p* < 0.01,  ^∗∗∗^ *p* < 0.001,  ^∗∗∗∗^ *p* < 0.0001; ns, not significant.

## 4. Discussion

Almost four decades ago, a reassortment event contributed to the emergence of very virulent IBDV (vvIBDV, A3B2) strains, which subsequently caused substantial economic losses in the global poultry industry [21]. Since then, IBDV has undergone extensive genetic evolution through point mutation, recombination and reassortment, resulting in considerable genetic diversity among circulating strains [22]. These changes may affect viral virulence [23, 24]. Although numerous studies have attempted to identify the viral determinants of IBDV virulence [25, 26], the precise molecular basis remains unclear. Both genome segments contribute to the pathogenic phenotype, and variation within the segment B‐encoded polymerase VP1 can modify virulence [27]. Consequently, in vivo studies remain essential for accurate pathotype determination. Historically, IBDV pathogenicity has been classified based on clinical signs, lesion and mortality in SPF White Leghorn chickens into variant, classical and very virulent pathotypes [28].

Recently, novel reassortant strains were detected in Poland [12], characterised by segment A derived from very virulent strains and segment B of unknown origin and classified as genotype A3B4 [22, 29]. A previous in vivo study demonstrated that A3B4 strains exhibit lower virulence than A3B2 strains in terms of mortality while retaining substantial pathogenic and immunosuppressive potential. Despite their reduced mortality, A3B4 strains successfully spread and become predominant in Poland [13], suggesting that factors other than acute virulence may contribute to their epidemiological fitness.

In the present study, multiple aspects of the pathogenesis of A3B4 Bug/03 and A3B2 75/11 strains were investigated, including viral replication in lymphoid and non‐lymphoid tissues, viral shedding, lymphoid organ development, body weight gain, immunosuppressive effects and host transcriptional responses.

Both IBDV strains significantly reduced body weight gain compared with the control group at selected time points (Figure 2). The A3B4 Bug/03 strain caused a transient reduction in body weight at 4 and 7 dpi (*p* = 0.0316 and *p* = 0.0104, respectively), followed by marked variability in body weight at 14 dpi, indicating uneven development among infected birds. Such heterogeneity may be relevant under field conditions, where impaired growth and reduced flock uniformity represent important economic consequences of IBDV infection [7]. In contrast, the A3B2 75/11 strain induced a more sustained reduction in body weight, with significant differences from controls at 7 and 14 dpi (*p* < 0.0001 and *p* = 0.0422, respectively). At 7 dpi, body weight was also significantly lower in the 75/11 group than in the Bug/03 group (*p* = 0.0007).

Both strains replicated efficiently in lymphoid and non‐lymphoid tissues. Although significantly higher viral RNA loads of 75/11 were detected at selected time points, including 2 and 7 dpi in the bursa and 7 dpi in the spleen, caecal tonsils and proventriculus, the overall tissue distribution and replication capacity of the two strains were broadly comparable (Figures 3 and 4). The highest viral loads for both strains were consistently detected in the bursa of Fabricius, confirming its role as the primary target organ for IBDV replication [30]. Notably, Bug/03 remained detectable for longer in several tissues despite slightly lower viral loads at some early time points. These findings indicate that acquisition of the heterologous B4 segment (segment B) does not substantially impair the ability of the A3B4 Bug/03 strain to replicate and disseminate within the host.

Interestingly, the kinetics of bursal damage differed between the two strains and were not directly associated with viral RNA load. A significant difference in BBIX between the Bug/03 and 75/11 groups was already detectable at 2 dpi, although the index remained above the threshold indicative of bursal atrophy in both groups. By 4 dpi, BBIX had decreased below 0.7 in Bug/03‐infected chickens, whereas it remained above this threshold in the 75/11 group. Thus, Bug/03 induced an earlier onset of measurable bursal atrophy despite comparable viral RNA loads of the two strains in the bursa at 4 dpi. In the 75/11‐infected chickens, bursal atrophy became evident from 7 dpi onward.

The significant difference in BBIX already detected at 2 dpi suggests that the divergence in the kinetics of bursal damage between the two strains was initiated very early after infection, before overt atrophy developed. This observation supports the possibility that the cellular events leading to lymphoid tissue loss in Bug/03‐infected bursae preceded the 4 dpi transcriptomic sampling point.

Early bursal atrophy has previously been described for antigenic variant strains [31, 32] and for ITA genotype A6B1 [33], indicating that the kinetics of bursal involution can differ markedly among IBDV strains. Importantly, histological lymphoid depletion and a gross reduction in bursal size do not necessarily occur simultaneously. Lupini et al. [33] observed severe follicular lymphocyte depletion as early as 2 dpi, while the bursae were still macroscopically enlarged; atrophy subsequently became evident at 4 dpi for the ITA strain and only at 7 dpi for the comparison strain. Likewise, Sharma et al. [32] demonstrated strain‐dependent differences between lymphoid depletion and the inflammatory component of bursal lesions: early plical atrophy associated with marked lymphoid depletion occurred in the variant strain, whereas the conventional strain induced prominent heterophilic infiltration and oedema. These observations indicate that gross bursal size during acute IBDV infection reflects not only lymphoid tissue loss but also concurrent oedema, exudation and inflammatory cell infiltration.

This distinction may be particularly relevant to the differences observed between Bug/03 and 75/11. One possible explanation is that Bug/03 caused an earlier net loss of lymphoid tissue, whereas a stronger or more prolonged inflammatory response to 75/11 transiently counterbalanced lymphoid depletion through oedema and cellular infiltration, thereby delaying the gross manifestation of atrophy. Such a mechanism would be consistent with the acute pathology of vvIBDV infection, in which substantial follicular depletion can coexist with bursal enlargement, oedema and haemorrhage during the early phase of infection [26]. Thus, the earlier decrease in bursal size after Bug/03 infection should not necessarily be interpreted as evidence of greater viral replication or greater overall tissue damage at this time point.

Analysis of the spleen‐to‐body weight ratios indicated significant splenic enlargement in both infected groups at 4 and 7 dpi compared with that in controls, with no significant differences detected at 2 or 14 dpi. These findings further indicate that the magnitude of lymphoid‐organ lesions was not simply proportional to viral RNA load. Macroscopic changes in lymphoid organs during acute IBDV infection result from a combination of virus‐induced lymphoid cell damage and host inflammatory responses [28], and strain‐dependent differences in these processes may therefore contribute to distinct pathological phenotypes [34].

Transcriptomic analysis of the bursa at 4 dpi provided additional evidence of strain‐dependent differences in the host response. Both viruses induced extensive transcriptional changes of a similar overall magnitude, but the functional organisation of these responses differed. Bug/03 infection was characterised predominantly by enrichment of pathways associated with ribosomal function, oxidative phosphorylation, RNA processing, cell‐cycle regulation and other metabolic and translational processes. Rather than indicating an absence of cellular injury, this profile may reflect substantial metabolic and translational reprogramming of the remaining bursal cell populations following early tissue damage. Similar alterations in cellular metabolic pathways have previously been reported during infection with classical IBDV strain [35]. However, whether such metabolic reprogramming contributes directly to the longer persistence of Bug/03 observed in some tissues cannot be determined from the present data.

In contrast, 75/11 induced a transcriptional profile more strongly associated with antiviral immunity, inflammatory signalling, B‐cell responses and apoptosis‐related processes, consistent with previous observations following infection with vvIBDV strains [36]. Examination of individual apoptosis‐related genes revealed; however, that cell‐death signalling was altered in both infected groups. In Bug/03‐infected bursae, increased expression was observed for several genes associated with death‐receptor signalling, including FASLG, TNFRSF10B, CASP10 and CASP18. Conversely, CASP9, AIFM3, GADD45B and DAPK2 showed stronger induction following 75/11 infection, while several genes with anti‐apoptotic functions were reduced in both groups. These findings therefore do not support the conclusion that Bug/03 induced a globally stronger apoptotic transcriptional response at 4 dpi. Rather, they suggest that the timing and mechanisms of cell death may differ between the two infections.

One possible explanation for the apparent discrepancy between the earlier bursal atrophy and the less prominent apoptosis‐related pathway enrichment in the Bug/03 group is that a substantial loss of susceptible B cells had already occurred before or by the 4 dpi sampling point. IBDV‐induced apoptosis can occur early during infection, and experimental studies have demonstrated rapid activation of apoptotic mechanisms in infected cells [37, 38]. Importantly, IBDV‐induced caspase activity is regulated at the protein level; activation of caspases 3 and 9 has been demonstrated through proteolytic activity during infection, meaning that transcript abundance alone cannot be used as a direct measure of ongoing apoptosis [37]. Indeed, CASP3 transcripts were decreased in both infected groups in the present dataset, which should therefore not be interpreted as evidence for the absence of caspase‐mediated cell death.

Because RNA‐seq was performed at 4 dpi, when bursal atrophy was already evident in Bug/03‐infected chickens, the transcriptional profile may represent a stage subsequent to the major loss of susceptible B cells rather than the peak phase of this process. Furthermore, bulk RNA‐seq reflects the cellular composition of the tissue at the time of sampling. Preferential depletion of B cells in the Bug/03‐infected bursa could therefore itself reduce the representation of transcriptional programmes associated with those cells, while increasing the relative contribution of surviving immune, stromal and epithelial cell populations. In this context, the enrichment of metabolic and translational pathways after Bug/03 infection may reflect the state of the remaining tissue rather than the molecular events responsible for initiating B‐cell loss. Conversely, the stronger apoptotic and inflammatory transcriptional signature after 75/11 infection may indicate that active cell death and immune‐mediated tissue injury were still ongoing at 4 dpi.

The possibility of an earlier wave of B‐cell apoptosis following Bug/03 infection is therefore biologically plausible but cannot be demonstrated by the present experiment. Direct confirmation would require earlier transcriptomic sampling and complementary assessment of apoptosis, for example, by TUNEL staining, detection of activated caspases or other cell‐death markers. This interpretation should therefore be regarded as a hypothesis rather than a demonstrated mechanism.

The distinct segment B background of Bug/03 may additionally contribute to the differences in the kinetics of bursal pathology. Reverse genetics studies have demonstrated that VP1 can influence IBDV pathogenicity independently of replication efficiency. Le Nouën et al. [27] showed that recombinant viruses with comparable replication levels nevertheless differed significantly in bursal atrophy and histological lesions depending on the composition of VP1 and specifically suggested that differences in early virus replication or host responses occurring before 4 dpi could contribute to these phenotypes. Similarly, Escaffre et al. [26] demonstrated that both genome segments contribute to vvIBDV pathogenicity. Therefore, the B4 segment of Bug/03 may have contributed to the distinct kinetics of virus–host interactions and bursal damage observed in the present study. However, because Bug/03 and 75/11 differ in both genome segments and are not isogenic viruses, the earlier bursal atrophy cannot be attributed specifically to segment B without further reverse‐genetics studies.

Taken together, these observations indicate that pathogenic outcome is not determined solely by viral replication efficiency. The comparatively small differences identified by direct transcriptomic comparison between the two infected groups suggest that Bug/03 and 75/11 activate a substantial shared host response but differ in the timing and relative contribution of inflammatory, apoptotic and metabolic processes. In Bug/03 infection, earlier lymphoid tissue loss may be followed by pronounced metabolic and translational reprogramming of the remaining bursal tissue, whereas in 75/11 infection, active immune‐mediated and apoptotic responses appear to remain prominent at 4 dpi. These differences may contribute to the distinct kinetics of tissue pathology observed between the strains.

Despite these differences in pathogenesis and host transcriptional responses, both strains induced substantial immunosuppression, as demonstrated by the reduced serological response to NDV vaccination and increased susceptibility to challenge. This indicates that the ability to interfere with host immune competence is retained by the A3B4 virus despite its lower mortality compared with that of the A3B2 strain. Thus, the divergent pathological outcomes of Bug/03 and 75/11 appear to reflect differences in the kinetics and nature of virus–host interactions rather than a simple difference in their overall immunosuppressive capacity.

## 5. Conclusions

In conclusion, the A3B4 Bug/03 strain displays a distinct pathogenic phenotype characterised by efficient replication in lymphoid and non‐lymphoid tissues, early bursal atrophy, prolonged detection in several tissues, substantial immunosuppressive effects and lower mortality than the A3B2 75/11 strain. The earlier bursal atrophy induced by Bug/03 despite comparable viral loads at 4 dpi highlights the importance of host–virus interactions, rather than viral replication alone, in determining tissue pathology. Transcriptomic data suggest that differences between Bug/03 and 75/11 primarily involve the timing and functional organisation of the host response, with metabolic and translational reprogramming predominating after Bug/03 infection and stronger inflammatory and apoptosis‐related responses persisting after 75/11 infection. However, an earlier wave of B‐cell death following Bug/03 infection remains a hypothesis that requires direct experimental confirmation. The ability of A3B4 viruses to combine efficient replication and marked immunosuppression with a less severe acute clinical presentation may favour their persistence and spread in poultry populations. These findings emphasise the need for continued surveillance of A3B4 IBDV strains and for further studies addressing the molecular mechanisms responsible for their distinct pathogenic phenotypes.

## Funding

The study was funded by the National Veterinary Research Institute (NVRI), Pulawy, Poland within the “NVRI Research Fund” awarded to Project Number F/113.

## Conflicts of Interest

The authors declare no conflicts of interest.

## Supporting Information

Additional supporting information can be found online in the Supporting Information section.

## Supporting information


**Supporting Information 1** Table S1: Differentially expressed genes (DEGs) identified by DESeq2 analysis across experimental groups.

**Supporting Information 2** Table S2: Functional enrichment analysis of differentially expressed genes (DEGs) identified between experimental groups using g:Profiler.

## Data Availability

The data that support the findings of this study are openly available in Sequence Read Archive (SRA) data at https://www.ncbi.nlm.nih.gov/sra, Reference Number PRJNA1463657.
